# Human airway model reveals host-pathogen co-infection dynamics of *Staphylococcus aureus* and *Pseudomonas aeruginosa*

**DOI:** 10.1093/ismeco/ycag284

**Published:** 2026-10-06

**Authors:** Katrine Madsen, Signe Lolle, Martin Saxtorph Bojer, Hanne Ingmer, Helle Krogh Johansen, Søren Molin, Ruggero La Rosa

**Affiliations:** The Novo Nordisk Foundation Center for Biosustainability, Technical University of Denmark, Kgs. Lyngby, 2800, Denmark; Department of Clinical Microbiology, Rigshospitalet, 2100 Copenhagen Ø, 9301, Denmark; Department of Veterinary and Animal Sciences, Faculty of Health and Medical Sciences, University of Copenhagen, Frederiksberg C, 1870, Denmark; Department of Veterinary and Animal Sciences, Faculty of Health and Medical Sciences, University of Copenhagen, Frederiksberg C, 1870, Denmark; Department of Clinical Microbiology, Rigshospitalet, 2100 Copenhagen Ø, 9301, Denmark; Department of Clinical Medicine, Faculty of Health and Medical Sciences, University of Copenhagen, Copenhagen, 2200, Denmark; Department of Health Technology, Technical University of Denmark, Kgs. Lyngby, 2800, Denmark; The Novo Nordisk Foundation Center for Biosustainability, Technical University of Denmark, Kgs. Lyngby, 2800, Denmark; Department of Clinical Microbiology, Rigshospitalet, 2100 Copenhagen Ø, 9301, Denmark; Department of Health Technology, Technical University of Denmark, Kgs. Lyngby, 2800, Denmark

**Keywords:** host-pathogen interactions, polymicrobial infections, *agr* quorum sensing, T6SS, virulence, cystic fibrosis, bacterial competition

## Abstract

Polymicrobial airway infections, particularly those involving *Staphylococcus aureus* and *Pseudomonas aeruginosa*, are a hallmark of severe chronic respiratory diseases such as those in people with cystic fibrosis (pwCF). Yet, the mechanisms enabling their co-existence remain poorly understood. Using a human air-liquid interface (ALI) airway model, we dissected host-pathogen and interspecies dynamics during mono- and co-infections with clinically relevant bacterial pathogens. We found that *S. aureus* strains exhibited distinct colonization patterns and virulence profiles, with the deletion of the *agr* quorum sensing locus leading to attenuated epithelial disruption. Additionally, the H1 component of *P. aeruginosa*’s type VI secretion system was critical for competitive exclusion of *S. aureus,* its deletion allowing for occasional co-localization of both species. Importantly, the order of co-infection strongly influenced pathogen dominance, with prior colonization of ALI models with *S. aureus* enabled coexistence with *P. aeruginosa*, thereby mimicking clinical observations in pwCF. Our results reveal that strain-specific traits and secretion systems influence microbial competition and persistence in the airway and underscore the importance of physiologically relevant models for studying chronic infections. Moreover, they provide the foundation for exploring therapeutic strategies for polymicrobial infections that account for the colonization sequence and microbial evolution for treatment design.

## Introduction

Multi-species microbial airway infections can cause severe exacerbation of pulmonary diseases, especially in people with cystic fibrosis (pwCF) [1–5]. Cystic fibrosis (CF) is characterized by dehydration of the airway secretion and excess mucus production, resulting in a microenvironment favoring the presence of certain bacterial species, including, but not limited to, *Staphylococcus aureus* and *Pseudomonas aeruginosa* [6–10]*. Staphylococcus aureus* is among the first pathogens to colonize the airways of young children with CF^11^. In most cases, *P. aeruginosa*, which is considered a later colonizer, displaces and outcompetes *S. aureus,* becoming the dominating airway pathogen [11]*.* This inverse correlation between *S. aureus* and *P. aeruginosa* during years of prolonged infection in the airways has been shown in several pwCF [12]. However, longitudinal analysis of pwCF airways infections has also shown that the two pathogens can co-exist within the same patient for extended periods of time [13]. This indicates that, albeit active competition between *S. aureus* and *P. aeruginosa* might be the primary driver of the observed anticorrelation, the two bacterial species can nonetheless co-exist in the airways of pwCF [14]. The presence of such contradictory findings highlights that the underlying mechanisms of *S. aureus–P. aeruginosa* interactions are still poorly understood. Importantly, *S. aureus* and *P. aeruginosa* co-existence can impact patient morbidity and mortality, which is of high clinical relevance [13]. Understanding of their colonization and infection dynamics, and the interspecies interactions within the host during infection, is, therefore, of high priority to optimize treatment and improve patients’ outcomes [4].

Competition between *S. aureus* and *P. aeruginosa* has been extensively studied in laboratory conditions *in vitro*, using both agar plates and liquid cultures, which do not resemble the *in vivo* host environment [15–17]. *P. aeruginosa*’s quorum sensing (QS) system has been shown to be a major regulatory system involved in *S. aureus–P. aeruginosa* interactions [15, 18]*.* In contrast, the potential role of the type VI secretion system (T6SS), which has largely been described as active only against Gram-negative bacteria, in mediating competitive interactions against the Gram-positive *S. aureus* remains largely unexplored [19, 20]. The H1-T6SS system delivers toxins such as the Tse1-Tse7 into neighboring bacterial cells, hereby disrupting and degrading membranes and cell walls of competing bacteria, along with interfering with their replication and growth [21–24]. Recently, it was shown that different isolates of *P. aeruginosa* sampled from the same individual at a single timepoint expressed different T6SS effectors. However, the number of effector genes did not influence the possibility that a strain could cause chronic infection [25]. By eliminating competing microbes, the T6SS provides *P. aeruginosa* a significant fitness advantage in multispecies communities, being an important weapon for dominating the ecological niche [17, 26, 27]. Therefore, changes in T6SS activity, along with broader adaptive evolutionary processes [28–31], may contribute to the observed *S. aureus* and *P. aeruginosa* interspecies dynamics. However, the molecular mechanisms governing their interactions *in vivo* remain poorly characterized.

In *S. aureus*, acquisition of loss-of-function mutations in the *agr* operon is frequently observed during long-term, persistent infections [32, 33] and can influence disease progression [34, 35]. Though the relevance and dynamics of *agr* dysfunction in chronic CF airway infections are context-dependent and may involve coexistence with *agr*-active subpopulations [36–39]. *agr* is the primary QS system in *S. aureus,* and it constitutes a major regulator of virulence, including the expression of a wide array of secreted toxins, enzymes, and QS molecules, many of which also target eukaryotic cells [40]. Its downregulation is often associated with a shift toward a more persistent, less immunogenic phenotype that favors long-term colonization over acute infection [32, 41, 42]. However, how different strains exert their virulence, including the role of Agr dysregulation in airway epithelial colonization and cytotoxicity, remains poorly characterized.

Recently, the development of new advanced infection model systems, such as the air-liquid interface (ALI) system, which recreates the physiology of the airway environment [43], has allowed the study of both *S. aureus* and *P. aeruginosa* infections in host-like conditions [29, 30,43–45]. However, their precise role in airway colonization during co-infection within the human host remains to be determined. Furthermore, the extent to which colonization patterns and interspecies microbial competition are strain-specific is still an open question. Addressing these points requires model systems that accurately recreate the host infection’s microenvironment and by employing clinically relevant microbial variants selected through within-host evolution. Importantly, understanding these interactions is not only critical for CF but may also be valuable for other chronic respiratory diseases characterized by polymicrobial infections.

Here, we investigated host–microbe interactions using an ALI model of human airway epithelium during infections with multiple *S. aureus* strains, including an *agr* mutant strain. This provided insight into strain-specific colonization patterns and specific host responses. Additionally, by co-infecting airway ALI models with both *S. aureus* and *P. aeruginosa,* we established a dual-species infection model system to investigate their competition and co-existence dynamics under *in vivo*-like conditions. Finally, we characterized the role of the *P. aeruginosa* H1-T6SS system in mediating co-existence and competition with *S. aureus* and its contribution to airway colonization. Altogether, our work sheds light on the infection capabilities of *S. aureus* and *P. aeruginosa* in polymicrobial infections, in a model system that closely mimics the human airway epithelium.

## Materials and methods

### Bacterial culture and strains

The following strains were used for the project: *S. aureus:* Newman, USA300(AH1263) [46], JE2, JE2*(Δagr),* and *P. aeruginosa* PAO1, PAO1(ΔH1-T6SS) [47]*.* Strains were routinely grown at 37°C, *S. aureus* on Tryptic Soy Broth (TSB) media and *P. aeruginosa* on lysogeny broth (LB) media.

### GFP and mKOk tagging of *P. aeruginosa*

*P. aeruginosa* strains were tagged either with a superfolder green fluorescent protein (sfGFP) or with mKusabira-Orange-kappa (mKOk) to enable visualization by confocal microscopy. Bacterial strains were tagged by four parental mating using the miniTn7 delivery plasmid (pJM220) [48, 49]. The employed plasmids contain either sfGFP or mKOK genes under the pTAC constitutive promoter. Transformants were selected on Pseudomonas isolation agar (PIA) supplemented with 30 μg/ml gentamicin. The final tagged strains were verified by fluorescence using a Leica DM4000 B epifluorescence microscope and growth rate was not affected by chromosomal tagging.

### Generation of JE2Δ*agr* strain

The *agr* deletion strain in JE2 was generated by phage transduction (phi11) of the deletion from *S. aureus* RN6911 as previously reported [50] and selection on tetracycline (5 mg/l). The strain was verified phenotypically by lack of hemolysis on blood plates compared to wildtype JE2, which is strongly hemolytic. Deficiency in autoinducing peptide production was confirmed by assaying ability of spent culture supernatant to induce a P3-*blaZ* reporter strain monitored by nitrocefin conversion [51]. The absence of the *agr* locus was confirmed by whole-genome sequencing.

### GFP tagging of *S. aureus* strains

*S. aureus* strains were marked with GFP by transformation with the pCM11 plasmid [46] and selection on erythromycin (10 mg/l). pCM11 encodes a codon-optimized variant of *gfp*, expressed by the constitutively active *sarA* promoter, thus providing strong fluorescence at all growth phases.

### Air-liquid interface model

ALI models we established using the BCi-NS1.1 cell line, which is a human telomerase reverse transcriptase (hTERT)-immortalized basal cell line originated from a healthy non-smoker donor [52]. BCi-NS1.1 seeded in Pneumacult™ - ExPlus Medium (STEMCELL Technologies) and expanded in T25 culture flasks in incubator 37C, 5% CO_2_, following manufacturers’ instruction. Confluent cells with a viability >80% were seeded (1.5^*^10^5^ cells/well) in collagen (Gibco) coated transwells (1.0 μm pore size; Falcon^®^). Cells were kept in Pneumacult™- ExPlus Medium until confluent and subsequently airlifted. 400 μl Pneumacult™ - ALI maintenance medium (STEMCELL Technologies) was added to the basolateral chamber and cells were left to differentiate for 28 days, with media change twice a week. Transepithelial electrical resistance (TEER) was measured once a week using EVOM2™ electrode. Mucus was removed once a week by PBS wash of apical compartment.

### Infection and characterization

Overnight cultures of bacteria were grown in 6 ml TSB or LB, respectively in 15 ml tubes while shaking and angled 45°. OD_600_ of the ON culture was measured and diluted to OD_600_ = 0.1 (infection culture) and left shaking in incubator at 37°C for 4–5 h. 1 ml of infection culture was centrifuged for 5 min. at 4500× *g,* the pellet was resuspended in D-PBS to reach OD_600_ = 1. The culture was then diluted to reach 1 × 10^5^ CFU/ml. 10 μl of the culture (10^3^ CFUs) was inoculated to the epithelial layer on the apical side at a MOI of 0.002. Transwells were incubated at 37°C with 5% CO_2_ for the indicated time (see individual experiments). Infection was terminated by adding 200 μl PBS to the apical side followed by TEER measurement using an EVOM2™ electrode. The ALI model was then immediately processed to collect bacteria from the different compartments.

### CFU, LDH, and IL-8 quantification

For colony forming unit (CFU) enumeration, cells were harvested from the apical and basolateral compartments. Bacteria from the apical compartment were suspended in PBS, while those in the basolateral side in ALI maintenance medium (Pneumacult™; STEMCELL Technologies). Bacteria were serially diluted in PBS and 10 μl of the serial dilutions was plated on LB agar plates. Bacteria attached to the epithelium were quantified by adding 200 μl PBS and scraping off the epithelium; samples were then quantified as described above. *S. aureus* and *P. aeruginosa* were discriminated on agar plates based on their morphology and growth phenotype.

From the infection, basolateral media was collected and used for lactate dehydrogenase (LDH) and interleukin-8 (IL-8) analysis. LDH was measured using The Invitrogen™ CyQUANT™ LDH Cytotoxicity Assay Kit (Cat. No. C20301) following manufacturers’ instruction. IL-8 was measured using Human IL-8/CXCL8 DuoSet ELISA kit (R&D Systems).

### Confocal microscopy analysis

Cells were fixated for confocal microscopy analysis using 4% paraformaldehyde (PFA) (in PBS). 400 μl PFA was added to the basolateral compartment and 200 μl to the apical compartment and left for 20 min. at 4°C. PFA was removed, and cells washed three times in D-PBS. Next, 400 μl blocking buffer (3% BSA, 1% saponin, 1% Triton-X in PBS, pH 7.4) was added to the basolateral compartment and 200 μl to the apical compartment and left for 30 min. at room temperature and washed three times with D-PBS. Primary antibodies were diluted in staining buffer (3% BSA, 1% saponin in PBS, pH 7.4) and 100 μl added to the apical part of the transwell and 400 μl staining buffer added to the basolateral compartment. Samples were left in the dark at room temperature for 2 h. Primary antibody and staining buffer were removed and transwells were washed three times for 5 min. in D-PBS. Antibodies included: DAPI diluted 1:500, Phalloidin 1:400 (Alexa Fluor). Samples were washed 3 times for 5 min in D-PBS and liquid was aspirated from the wells. Transwell filters were cut out using a scalpel and transferred to a microscope slide. 8 μl VECTASHIELD® Antifade Mounting Medium (VWR, VECTH-1000) was added on top of the sample and sealed with a glass slide and nail polish. Microscopy slides were visualized with a Leica Stellaris 8 Confocal Microscope (40×, 1.3 oil) and analyzed using ImageJ software.

### Whole-genome sequencing of *Δagr* and ΔH1-T6SS mutant strains

The genomes of the recombinant strains *S. aureus Δagr* and *P. aeruginosa* ΔH1-T6SS was sequenced to confirm the presence of the specific mutations. gDNA extraction, library preparation and sequencing were performed by Genewiz (DE). Briefly, gDNA was extracted from cell pellets using QIAamp DNA Mini Kit following manufacturer’s instructions (Qiagen, Hilden, Germany). DNA libraries were prepared using the NEBNext Ultra II DNA Library Preparation kit accordingly to the manufacture’s recommendations (NEB, Ipswich, MA, USA). gDNA was fragmented by shearing with a Covaris instrument, end repaired and adenylated. Adapters were ligated after adenylation of the 3’ ends followed by enrichment by limited-cycle polymerase chain reaction (PCR). Adapter-ligated DNA libraries were cleaned up and validated using Agilent TapeStation and quantified using Qubit 2.0 Fluorometer. Libraries were sequenced on an Illumina NovaSeq XPlus instrument according to the manufacturer’s instructions. Samples were sequenced using a 2 × 150 paired-end (PE) configuration. Sequencing reads were trimmed, and low-quality reads and potential contamination from adapters were removed using Trimmomatic (v 0.40) tool [53]. Genomic analysis was conducted by Breseq v0.40.1 [54] using default settings to identify genomic variants relative to respectively JE2 reference genome (NCBI: NZ_CP020619.1) and PAO1 reference genome (NCBI: NC_002516.2). The identified mutations were further confirmed using the Integrative Genomics Viewer (IGV) software v2.19.8.

### Statistical analyses

GraphPad Prism 10.4.1 was used for statistical analysis. ALI data were represented as mean ± standard error of the mean (SEM). Biological replicates represent independent experiments performed with cells from different passages and technical replicates cells from same passage. Statistical comparisons used included calculated using one-way analysis of variance (ANOVA) and two-way ANOVA with Tukey’s post hoc test. *P*-value <.05 was considered statistically significant. All figures were completed in Adobe Illustrator Artwork 28.7.10. All data are provided within the manuscript as Supplementary Data 1.

## Results

### Strain-specific *S. aureus* infection patterns in ALI models

To investigate potential differences in the colonization patterns of distinct *S. aureus* strains in conditions that mimic the human lung epithelium, we performed infection assays in ALI infection models based on the BCi-NS1.1 cell line, a hTERT-immortalized basal cell line derived from a healthy non-smoker [52]. ALI models differentiate into a human airway epithelium representative of the *in vivo* cell composition and physiological conditions, with the apical side exposed to air and the basolateral side containing the growth medium [52]. We performed colonization studies employing the *S. aureus* strains Newman, USA300(AH1263) [55] and JE2 [56]. The Newman strain has been isolated from a human infection and shows a robust virulence phenotype [57]. The USA300 is a hypervirulent strain due to high secretion of α-toxins known to damage epithelial tight-junctions, increased *agr-*response, and production of QS-regulated toxins [58]. Because of its widespread use in laboratories and available tools for mutagenesis, we also included the USA300 derivative JE2 strain, which exhibit a hypervirulent phenotype similar to USA300 [56, 58].

To determine the systemic response of the host tissue, we monitored three tissue-specific indicators of the infections: transepithelial electrical resistance (TEER), lactate dehydrogenase (LDH) release, and interleukin-8 (IL-8) secretion. TEER quantifies the epithelial layer integrity, and its decrease upon infection indicates epithelial disruption. The release of LDH indicates damage of the epithelial plasma membranes and quantifies cellular cytotoxicity of cells undergoing damage and death. IL-8 is a proinflammatory cytokine released by epithelial cells upon infection, which plays an important role in immune cell recruitment independently of their presence/absence in the ALI models.

To evaluate the dynamics of host colonization, we compared changes in TEER at 16 and 24 h post infection (hpi) relative to baseline (0 hpi). Overall, TEER decreased gradually over time for all strains, consistent with their virulence profile (Fig. 1A). At 16 hpi, the Newman strain showed only minor reduction in TEER, while the USA300 and JE2 strains, showed a more pronounced decrease, with TEER values already dropping to around 0.6 and 0.7, respectively. At 24 hpi, TEER decrease was substantial for all strains (Newman = 0.39 ± 0.070; JE2 = 0.26 ± 0.017), with the USA300 strain exhibiting the greatest TEER drop (0.14 ± 0.038) (Fig. 1A). For all strains, we observed an increase in LDH release at 24 hpi compared to 16 hpi (Two-way ANOVA with Tukey’s post hoc test, p < 0.01). Moreover at 24 hpi, the USA300 strain caused significantly higher LDH release relative to both Newman and JE2 infections (Two-way ANOVA with Tukey’s post hoc test, *P* < .01) (Fig. 1B). Similarly, we observed an increase in IL-8 secretion at 24 hpi compared to 16 hpi (two-way ANOVA with Tukey’s post hoc test, *P* < .0001), confirming a strong immune response of the epithelium upon infection (Fig. 1C). However, no differences were observed between strains. Bacterial growth was quantified at 16 hpi and 24 hpi in the apical and basolateral compartments of the ALI model, as well as from bacteria attached to the epithelium [59]. At 16 hpi, strain showed no differences across compartments (two-way ANOVA with Tukey’s post hoc test, *P* > .05), and no CFUs were detected basolaterally, indicating no bacterial epithelial penetration (Fig. 1D). At 24 hpi, significantly more USA300 CFUs were measured in the apical compartment compared to both Newman and JE2 (two-way ANOVA with Tukey’s post hoc test, *P* < .0001). Both USA300 and JE2 strains were found attached to the epithelium at significantly higher CFUs compared to the Newman strain (two-way ANOVA with Tukey’s post hoc test, *P* <.05). At 24 hpi, no bacteria were detected in the basolateral compartment following infection with the Newman strain. In contrast, basolateral bacterial counts reached 2 × 10^4^ CFU for USA300 and 2 × 10^2^ CFU for JE2, indicating strain-dependent epithelial penetration and confirming the hypervirulent phenotype of strains USA300 and JE2 (Fig. 1D). Confocal microscopy analysis revealed that at 16 hpi, all strains exhibited a similar colonization pattern with bacteria mostly dispersed as single cells across the epithelial surface (Fig. 1E). However, at 24 hpi, the USA300 and JE2 were found dispersed across the epithelium, whereas the Newman strain formed localized bigger colonies on top of the epithelium (Fig. 1E).

**Figure 1:**
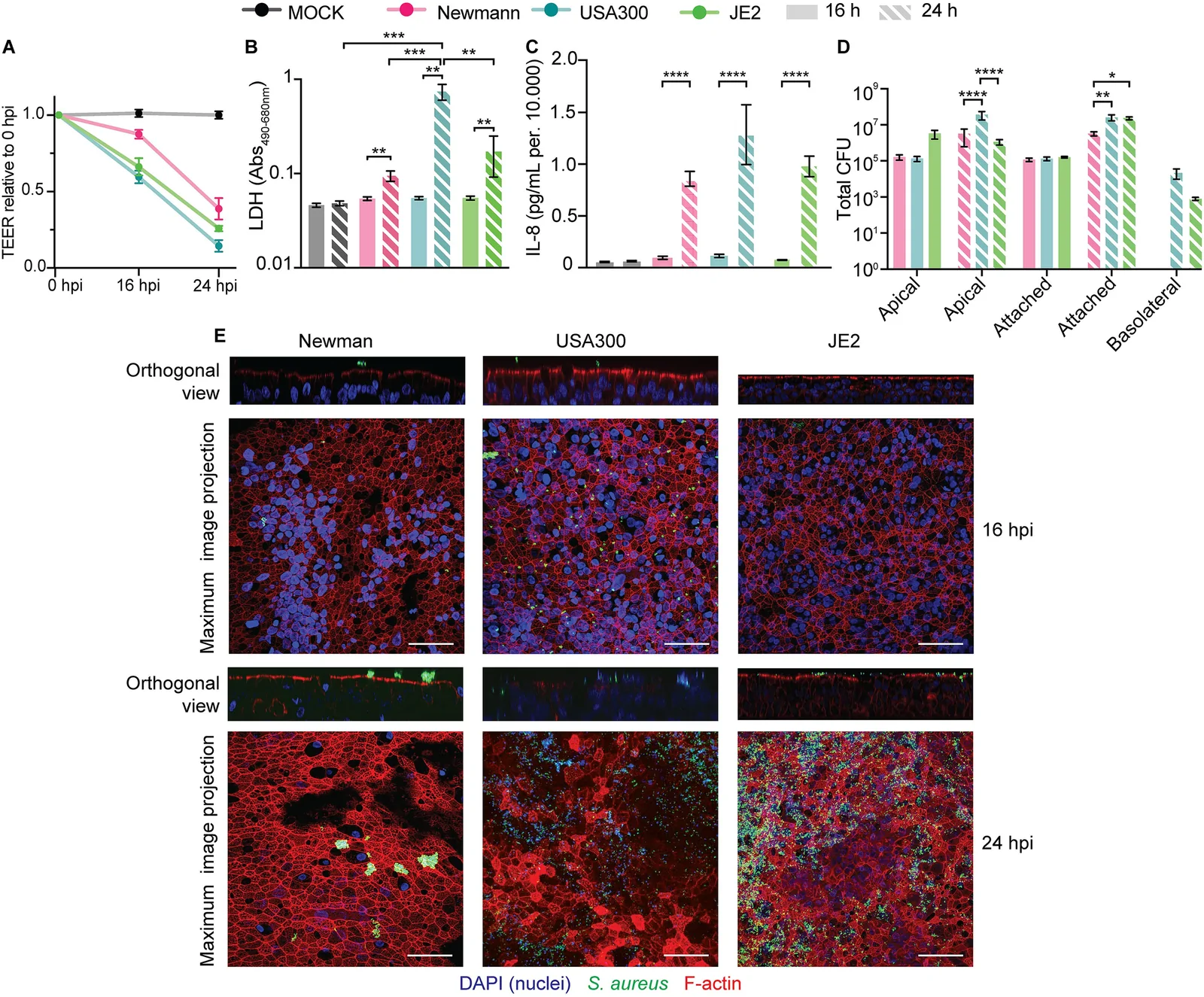
Strain-specific *S. aureus* infection patterns in ALI infection models. (A) TEER at 16 h post-infection (hpi) and 24 hpi relative to 0 hpi. (B) LDH release measured as Abs_490–680_. (C) IL-8 release measured in pg/ml. (D) Bacterial growth (total CFU) in the apical and basolateral compartment and attached to the epithelium calculated from 200 μl recovered samples. Infections were performed at 16 and 24 h post infection (hpi); unless noted, 16 hpi is shown with solid bars and 24 hpi with dashed bars. In grey mock uninfected epithelial cells, in pink Newman strain, in blue USA300 strain, and in green JE2 strain. (E) Confocal microscopy images of USA300, Newman and JE2 infections at 16 hpi and 24 hpi. In blue (DAPI) human nuclei, in red (phalloidin RFP) F-actin and in green (GFP) *S. aureus*. Images were acquired at 40× and scale bar = 50 μm. In all cases, data are shown as mean ± SEM of three independent biological replicates. Statistical significance was determined by two-way ANOVA with Tukey’s post hoc test where ^*^P <.05, ^**^P <.01, ^***^P <.001, ^****^P <.0001.

These results indicate that *S. aureus* strains exhibit distinct colonization patterns and host interaction profiles in ALI models, highlighting the importance of strain-specific characteristics in epithelial disruption, immune activation, and tissue invasion.

### *agr* mutation suppresses *S. aureus* virulence toward the airway epithelium

Agr is a major regulator of QS and virulence in *S. aureus* [41, 42], and we hypothesized a change in infection dynamics as a consequence of its disruption. For this, we investigated the colonization capacity of the JE2 strain and its isogenic *Δagr* mutant strain in ALI infection models.

At 24 hpi, the JE2 strain caused a TEER decline to around 0.3-fold, whereas the TEER remained largely unchanged for the *Δagr* infections compared to 0 hpi (one-way ANOVA with Tukey’s post hoc test, *P* <.0001) (Fig. 2A). This lower degree of disruption of the epithelial layer by the *Δagr* mutant was further supported by LDH measurements, where the JE2 infections caused a substantially higher release of LDH compared to infection with the *Δagr* strain and mock (one-way ANOVA with Tukey’s post hoc test, *P* <.05) (Fig. 2B). No significant difference in IL-8 release was observed between JE2 and *Δagr* strains indicating bacterial recognition by the epithelium despite the mutation (one-way ANOVA with Tukey’s post hoc test, p < 0.0001) (Fig. 2C). Equal number of CFUs were found in the apical side, whereas significantly more JE2 were found attached to the epithelium compared to the *Δagr* (two-way ANOVA with Tukey’s post hoc test, *P* <.0001) (Fig. 2D). Notably, only the JE2 strain was able to penetrate the epithelial layer as evidenced by its presence in the basolateral compartment (Fig. 2D). Importantly, growth profiling in TSB medium revealed similar doubling times for both strains (Δ*agr*: 0.85 h^−1^ ± 0.02; JE2: 0.89 h^−1^ ± 0.02) as previously reported [60], suggesting that differences in colonization patterns between strains are likely driven by differences in host-pathogen interactions rather than their growth profiles. Confocal microscopy analyses showed that at 24 hpi, the JE2 strain was scattered on top of the epithelial layer (Fig. 2E). On the contrary, the *Δagr* strain was localized in condensed colonies on top of the epithelium, and no penetration was observed (Fig. 2E).

**Figure 2:**
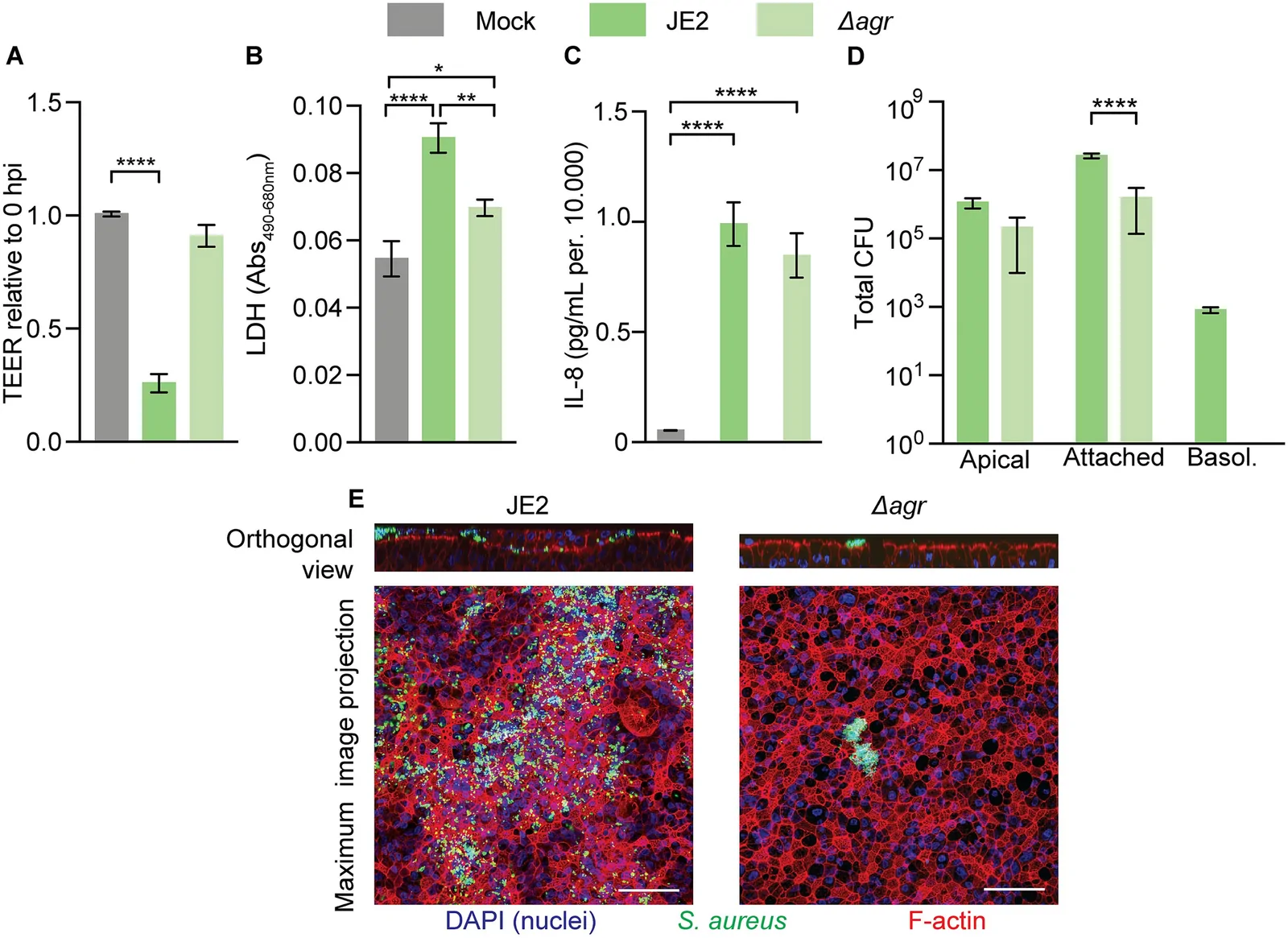
Effects of *agr* mutation on *S. aureus* colonization and virulence. (A) TEER of mock, JE2 and *Δagr* at 24 h post-infection (hpi) relative to 0 hpi. (B) LDH release measured as Abs_490–680_. (C) IL-8 release measured in pg/ml. (D) Bacterial growth (total CFU) in the apical and basolateral compartment and attached to the epithelium calculated from 200 μl recovered samples. (E) Confocal microscopy images of JE2 and *Δagr* at 24 hpi. In blue (DAPI) human nuclei, in red (phalloidin RFP) F-actin and in green (GFP) *S. aureus*. Images were acquired at 40× and scale bar = 50 μm. In all cases, data are shown as mean ± SEM of three independent biological replicates. Statistical significance was determined by one-way (A, B, C) or two-way (D) ANOVA with Tukey’s post hoc test, where ^*^P < .05, ^**^P < .01, ^****^P < .0001.

These results confirm that disruption of the *agr* system significantly attenuates *S. aureus* virulence in ALI models, leading to reduced epithelial damage, limited tissue invasion, and altered colonization dynamics.

### Impact of H1-T6SS deletion on virulence and epithelial interaction in ALI infection models

The T6SS is a contact-dependent structure used by *P. aeruginosa* to inject effector proteins into neighboring cells, contributing to host cell interactions and interbacterial competition [27]. We analyzed the infection capabilities of a H1-T6SS deletion strain, as it is the primary system responsible for interspecies antagonism, in contrast to H2- and H3-T6SS, which are involved in host cell invasion and interactions [61].

We infected ALI models with *P. aeruginosa* PAO1 wild type and the isogenic ΔH1-T6SS derivative [47], and evaluated host responses at 14 hpi in absence of other bacterial competitors. Both strains caused a significant drop in TEER (Fig. 3A), and increased LDH release (Fig. 3B) compared to the mock, with no significant differences between the strains (one-way ANOVA with Tukey’s post hoc test, *P* <.001). Infections with both PAO1 and ΔH1-T6SS elicited an immune response with IL-8 secretion slightly higher for PAO1 relative to ΔH1-T6SS (one-way ANOVA with Tukey’s post hoc test, *P* <.05; Fig. 3C). Comparing the CFUs between the two strains did not reveal any significant differences in the apical and attached compartments (one-way ANOVA with Tukey’s post hoc test, *P* >.05). However, we observed significantly more CFUs for the ΔH1-T6SS strain in the basolateral compartment relative to PAO1 (two-way ANOVA with Tukey’s post hoc test, *P* <.001) (Fig. 3D). For both strains, confocal microscopy analysis revealed the presence of large colonies on top of the epithelium for which we did not observe any substantial differences (Fig. 3E).

**Figure 3:**
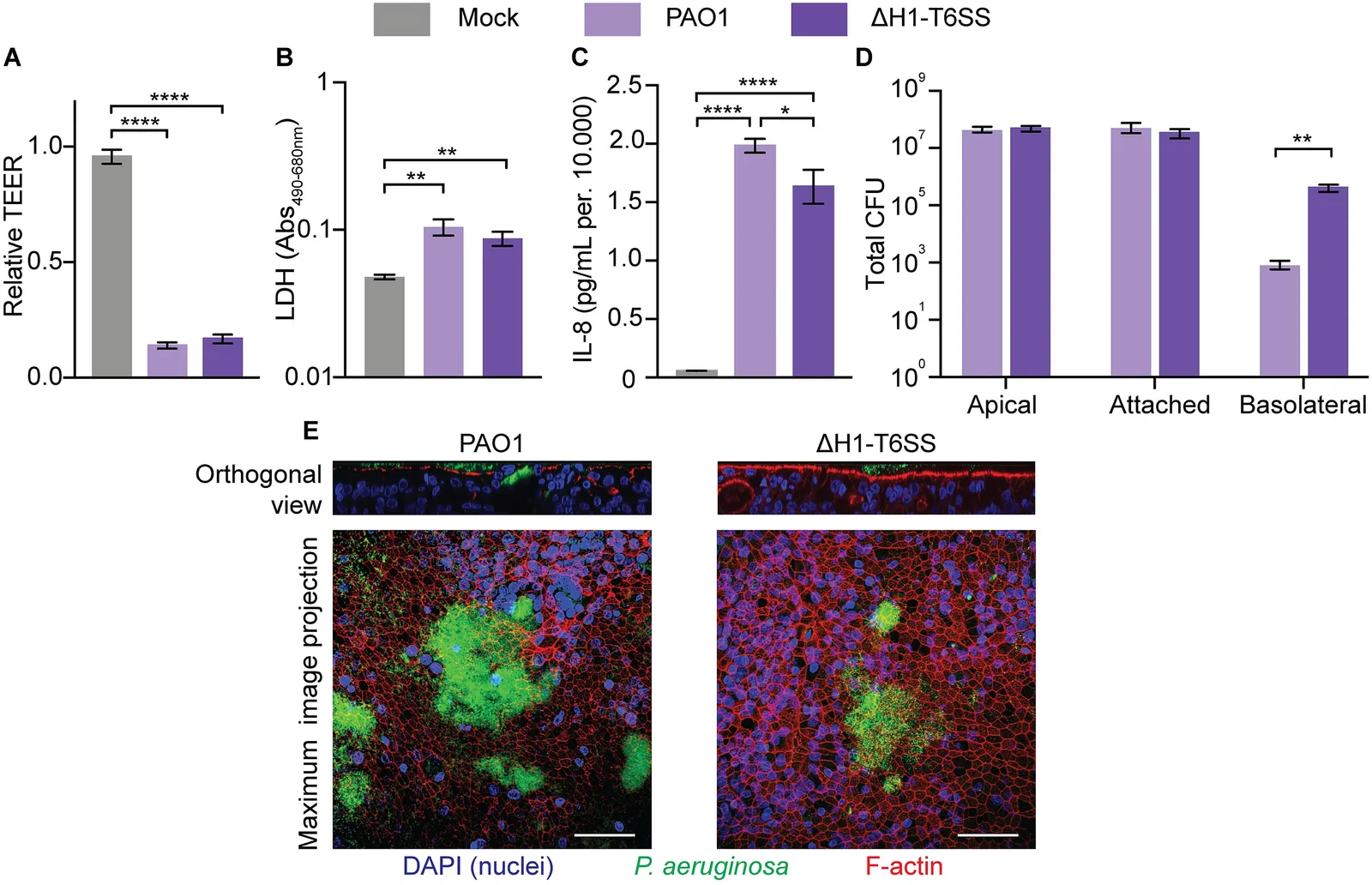
Infection phenotype of PAO1 and ΔH1-T6SS in ALI models. (A) TEER of mock, PAO1 and ΔH1-T6SS at 14 h post-infection (hpi). (B) LDH release measured as Abs_490–680_. (C) IL-8 release measured in pg/ml. (D) Bacterial growth (total CFU) in the apical and basolateral compartment and attached to the epithelium calculated from 200 μl recovered samples. (E) Confocal microscopy images of PAO1 and ΔH1-T6SS. In blue (DAPI) human nuclei, in red (phalloidin RFP) F-actin and in green (GFP) *P. aeruginosa*. Images were acquired at 40× and scale bar = 50 μm. In all cases, data are shown as mean ± SEM of three independent biological replicates. Statistical significance was determined by one-way (A, B, C) or two-way (D) ANOVA with Tukey’s post hoc test, where ^*^P < .05, ^**^P < .01, ^****^P < .0001.

These results suggest that deletion of the H1-T6SS does not modify epithelial damage or immune activation in ALI models but might promote epithelial penetration. Since no difference in bacterial growth rate (ΔH1-T6SS = 1.2 h^−1^ ± 0.05; PAO1 = 1.1 h^−1^ ± 0.03) was observed neither in laboratory conditions nor in ALI infection (apical and attached compartment), this result suggests a potential additional role of the H1-T6SS in modulating host interactions.

### Colonization order and H1-T6SS function modulate *S. aureus–P. aeruginosa* interactions and host responses in airway epithelium

To investigate how colonization timing and order influence interspecies dynamics and host responses, we performed co-infection assays in ALI models using *S. aureus* and *P. aeruginosa* either added simultaneously, as commonly done *in vitro,* or sequentially with *S. aureus* preceding *P. aeruginosa* to mimic the typical colonization pattern in pwCF.

When either USA300 or Newman strain were added simultaneously in equal amounts with PAO1 in the ALI model, PAO1 outcompeted both strains, leaving close to no visible *S. aureus* growth at the end of the infection and only PAO1 was recovered in the ALI compartments (Fig. S1). In contrast, when the JE2 strain was allowed to colonize the ALI model for 24 h prior to the addition of PAO1 and the co-infection was analyzed after 14 h, equal amounts of bacteria were recovered for both pathogens both in the apical compartment and attached to the epithelium (two-way ANOVA with Tukey’s post hoc test, *P* >.05) (Fig. 4A, see JE2 (PAO1 co-culture) *vs* PAO1 (JE2 co-culture)). Only *P. aeruginosa* was recovered from the basolateral compartment (Fig. S2A), which is consistent with previous findings showing its ability to outcompete *S. aureus* in liquid cultures [62]. Similar results were obtained for co-infected cultures of either *S. aureus* Newman or USA300 with *P. aeruginosa*, underlining the importance of the order of colonization despite intrinsic differences between *S. aureus* strains (Fig. S3). Prolonged co-infection times with *S. aureus* and *P. aeruginosa* resulted in severely damaged epithelium, and no reliable quantitative measurements could be obtained. The host responses to the co-infections (TEER, LDH and IL-8) were comparable to those observed for infections with PAO1 alone and exacerbated relative to JE2 single infection (one-way ANOVA with Tukey’s post hoc test, *P* <.05) (Fig. 4B-D). The presence of both pathogens was supported by confocal microscopy analysis showing that both *S. aureus* (in green) and *P. aeruginosa* (in red) were homogeneously distributed and co-colonized the airway epithelium (Fig. 4E).

**Figure 4:**
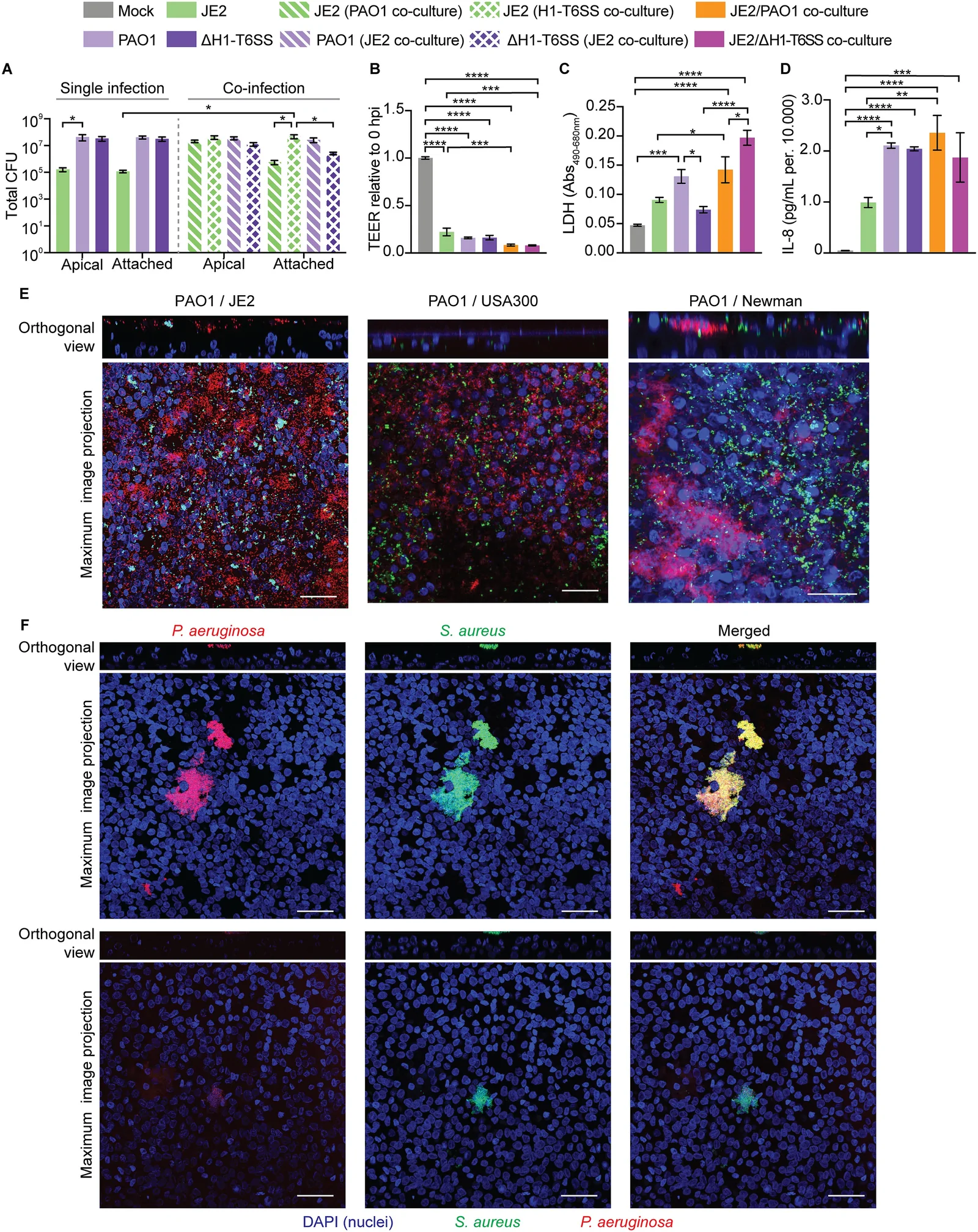
Dynamics of *S. aureus* and *P. aeruginosa* co-infection. (A) Bacterial growth (total CFU) in the apical and basolateral compartment and attached to the epithelium, calculated from 200 μl recovered samples, at 38 h post-infection (hpi) (24 h *S. aureus* pre-incubation plus 14 h of *P. aeruginosa* co-infection). (B) TEER. (C) LDH release measured as Abs_490–680_. (D) IL-8 release measured in pg/ml. (E-F) confocal microscopy images of *S. aureus* and *P. aeruginosa* in co-infection. In blue (DAPI) human nuclei, in red (RFP) *P. aeruginosa*, and in green (GFP) *S. aureus*. Images were acquired at 40× and scale bar = 50 μm. In (F), images show examples of micro-colonies of *S. aureus* and *P. aeruginosa* ΔH1-T6SS where both species are colocalized. In all cases, data are shown as mean ± SEM of three independent biological replicates. Statistical significance was determined by one-way (B, C, D) or two-way (A) ANOVA with Tukey’s post hoc test where ^*^P < .05, ^**^P < .01, ^***^P < .001, ^****^P < .0001.

Since the T6SS of *P. aeruginosa* is directly involved in interspecies competition and based on recent data suggesting a broader role of the T6SS in *S. aureus–P. aeruginosa* interactions [19], we co-infected the ALI model with the JE2 strain and the ΔH1-T6SS mutant strain. Bacterial growth analysis revealed significantly more JE2 strain attached to the epithelium when co-infected with the ΔH1-T6SS mutant strains compared to PAO1 (two-way ANOVA with Tukey’s post hoc test, *P* <.05) (Fig. 4A). TEER drop, LDH release and IL-8 secretion were, instead, comparable between co-infections with a slightly higher LDH release for ΔH1-T6SS strain co-infection (one-way ANOVA with Tukey’s post hoc test, *P* <.05) (Fig. 4B-D). Confocal microscopy analysis showed that the colonization patterns were generally comparable to the co-infection with PAO1 and JE2 (Fig. S2B). However, thorough analysis across several samples revealed confined colonies consisting of both pathogens, suggesting the possibility of co-colony formation when *P. aeruginosa* lacks a functioning H1-T6SS (Fig. 4F and S2A).

Together, these results show that colonization order and the presence of a functional H1-T6SS partly shape the outcome of *S. aureus* and *P. aeruginosa* interactions in airway epithelium, influencing both bacterial distribution and host responses.

## Discussion

Polymicrobial infections are often associated with increased morbidity and mortality in several diseases. In the airways, *S. aureus* and *P. aeruginosa* frequently co-exist, yet the interactions governing their coexistence and competitive dominance remain poorly understood. In this study, we used a human ALI airway model to investigate the dynamics of *S. aureus* and *P. aeruginosa* co-infection and species dominance.

In pwCF, it has been shown that either *S. aureus* dominantly colonizes the airways for several years [26], before being gradually substituted by *P. aeruginosa*, driven by competitive interactions between the two species [10], or that both pathogens co-exist long-term [13]. However, while the clinical impact of *S. aureus*–*P. aeruginosa* co-infection on disease progression is well established, molecular mechanisms underlying competition, co-existence and infection dynamics between *S. aureus* and *P. aeruginosa* are still not fully understood. In our *in vitro* experimental setup resembling the airway environment, we found that the order in which *S. aureus* and *P. aeruginosa* infect airway epithelial cells plays a critical role in determining species dominance. Indeed, while competition dominated when both species were added simultaneously to the ALI model, with *P. aeruginosa* being dominant, prior infection with *S. aureus* allowed both species to coexist throughout the course of the experiment. Early colonization may, indeed, allow a pathogen to modify the epithelial surface, compete for nutrients, and trigger host responses that may inhibit or facilitate subsequent colonization by another species. For example, *P. aeruginosa* introduced first may produce antimicrobial compounds or disrupt epithelial integrity, limiting *S. aureus* colonization, whereas *S. aureus* arriving first may establish protective niches that resist displacement [63]. Clinically, this mirrors observations in pwCF, where *S. aureus* often colonizes early in life, with *P. aeruginosa* taking over in later stages [63]. Unlike conventional liquid co-cultures, where *P. aeruginosa* invariably dominates [62], the ALI model reveals that the spatial architecture of the airway epithelium provides niches that can sustain *S. aureus* persistence during co-infection. Nevertheless, while our data support the hypothesis that the order of colonization may influence pathogen dominance, in patients this effect may depend on additional factors, such as the immune response, underlying conditions, concurrent treatments, and length of infection, which are not captured in the ALI infection model. Moreover, although our data demonstrate that unrelated, well-defined non-CF reference strains and isogenic mutants can reproduce co-infection patterns relevant to CF-like infection dynamics, these strains do not fully capture the phenotypic and genetic diversity of clinical CF isolates. Validation using clinical isolates, particularly co-isolated *S. aureus*–*P. aeruginosa* pairs, will therefore be required to establish the clinical relevance and generalizability of our findings.

*In vitro* studies have demonstrated that *P. aeruginosa* produces several virulence factors regulated by QS, such as pyocyanin, rhamnolipids, and HQNO (2-heptyl-4-hydroxyquinoline N-oxide), that inhibit *S. aureus* [64]. In response to these, *S. aureus* can adapt by switching to small colony variants, a phenotype characterized by slow growth, reduced metabolism, and increased resistance to oxidative stress and antibiotics, which facilitates persistence under such conditions [65]. While the role of QS in *P. aeruginosa* is well characterized, the activity of the T6SS in Gram-positive/Gram-negative interactions remains poorly characterized due to the assumption that the T6SS is mainly active against Gram-negative species. However, recent studies suggest that the T6SS may contribute to shaping bacterial community composition within the human microbiota and in polymicrobial infections, even against Gram-positive species [16, 19]. Interestingly, co-infection of the ALI model with the H1-T6SS mutant strain allowed for a higher *S. aureus* count due to the reduced interspecies competitive advantage of *P. aeruginosa* [20–23]*.* Additionally, we identified some confined microcolonies where both species were present, in contrast to the otherwise dispersed colonization patterns observed with the wild-type PAO1. This phenomenon was primarily mediated by the lack of a functional T6SS rather than controlled by QS. This suggests that mutations in the H1-T6SS could facilitate the occasional formation of stable co-infection microcolonies by preventing *S. aureus* killing, antagonism, and *P. aeruginosa* dominance. These results are in agreement with earlier hypotheses and suggest a broader role for the T6SS [16, 19]. Importantly, *P. aeruginosa* undergoes extensive within-host adaptation during chronic CF infections, including loss of virulence-associated functions [66, 67]. Our observation that deletion of H1-T6SS facilitates *S. aureus* persistence and co-colony formation suggests that evolutionary loss of T6SS activity in chronically adapted *P. aeruginosa* strains could be one mechanism enabling the long-term coexistence observed in some pwCF [13]. The co-localized microcolonies observed in the ΔH1-T6SS co-infections may additionally reflect a spatial organization relevant to polymicrobial biofilms within the CF airway. However, further studies are needed to further investigate the involvement of H1-T6SS within co-infected individuals.

Beyond its role in interspecies competition, the H1-T6SS also appeared to influence host–pathogen interactions during mono-infection. Indeed, deletion of H1-T6SS resulted in significantly higher basolateral bacterial counts in mono-infection, despite comparable apical growth and epithelial damage. This suggests a potential role for the H1-T6SS in modulating host–pathogen interactions, possibly through effects on epithelial barrier integrity or host cell invasion pathways. However, this finding warrants further investigation, as it may have implications for understanding how T6SS loss during chronic adaptation affects tissue invasion.

*S. aureus* strains showed marked differences in ALI models linked to their virulence profile. The Newman strain formed localized colonies, exhibited reduced cytotoxicity, and was unable to cross the epithelial barrier, whereas the USA300 and JE2 strains were dispersed across the epithelial surface and penetrated the epithelial barrier. These distinct colonization patterns suggest that strain-specific virulence profiles directly shape the spatial dynamics of airway colonization. This has important implications for clinical infections, where the infecting *S. aureus* genotype may determine whether the infection remains localized or becomes invasive, and how it interacts with co-infecting species. Additionally, mutation of the *agr* regulator fully attenuated the virulence of *S. aureus* by changing the bacterial colonization capacity, reducing epithelial damage, and limiting immune cell recruitment. This is in line with the presence of *agr*-defective isolates in patients with pneumonia [32, 33, 68]. Recent works suggest that elevated levels of sialic acid may confer a fitness advantage to *agr* mutants, suggesting that the reduced epithelial damage observed in ALI models may represent only one aspect of the benefits associated with *agr* dysfunction [26]. However, further investigations are required to fully characterize the adaptive advantages conferred by *agr* inactivation [69].

Importantly, co-infection with *S. aureus* and *P. aeruginosa* can modulate antibiotic susceptibility, either increasing or decreasing resistance, highlighting the complexity of interspecies interactions [70–72]. Poor correlation between *in vitro* susceptibility and clinical response remains a major challenge [73]. Therefore, testing antibiotics in multispecies, human-relevant models may improve therapeutic strategies for polymicrobial infections [29, 31], providing additional insight into treatment failure, host responses, and infection dynamics.

Altogether, our results demonstrate that colonization order, strain-specific virulence, and H1-T6SS function collectively shape the outcome of *S. aureus*–*P. aeruginosa* co-infection in a physiologically relevant airway model. These findings provide mechanistic insight into the clinical observations of species competition and coexistence in pwCF and underscore the critical role of the airway epithelium in modulating infection outcomes.

## Supplementary Material

Supplementary_material_ycag284

## Data Availability

The datasets generated during and/or analyzed during the current study are available within the paper in the Supplementary Data 1 and 2. Whole genome sequencing raw data of the recombinant strains is publicly available through the SRA number PRJNA1478433.
